# On Mammalian Totipotency: What Is the Molecular Underpinning for the Totipotency of Zygote?

**DOI:** 10.1089/scd.2019.0057

**Published:** 2019-07-16

**Authors:** Kejin Hu

**Affiliations:** Department of Biochemistry and Molecular Genetics, University of Alabama at Birmingham, Birmingham, Alabama.

**Keywords:** zygote, totipotency, embryogenesis, embryonic stem cells, reprogramming, blastomere, epigenetic totipotency

## Abstract

The mammalian zygote is described as a totipotent cell in the literature, but this characterization is elusive ignoring the molecular underpinnings. Totipotency can connote genetic totipotency, epigenetic totipotency, or the reprogramming capacity of a cell to epigenetic totipotency. Here, the implications of these concepts are discussed in the context of the properties of the zygote. Although genetically totipotent as any diploid somatic cell is, a zygote seems not totipotent transcriptionally, epigenetically, or functionally. Yet, a zygote may retain most of the key factors from its parental oocyte to reprogram an implanted differentiated genome or the zygote genome toward totipotency. This totipotent reprogramming process may extend to blastomeres in the two-cell-stage embryo. Thus, a revised alternative model of mammalian cellular totipotency is proposed, in which an epigenetically totipotent cell exists after the major embryonic genome activation and before the separation of the first two embryonic lineages.

## Introduction

As a critical starting point of a mammalian life, the zygote is described in the literature as being totipotent [1–4]. Ostensibly, this characterization seems informative, considering that a zygote eventually leads to the formation of all types of cells within an individual as well as all the extraembryonic cells supporting the development of the embryo proper and fetus. However, close consideration of the implications of totipotency in juxtaposition with the properties of a zygote calls into question whether a zygote is truly totipotent and whether the use of this term is indeed accurate.

This essay first defines three types of totipotency on the basis of the molecular underpinnings: genetic, epigenetic, and the maternally derived biochemical totipotency. My clarification of different totipotent concepts leads to arguments to support a suggestion that the mammalian zygote is not totipotent transcriptionally, epigenetically, and functionally although it is totipotent genetically as any other diploid cells are. Yet, this essay suggests that the zygote retains significant totipotent reprogramming factors from its parental oocyte. Finally, I propose that some mouse blastomeres, if not all, from a four-cell embryo, or from an early eight-cell embryo before its compaction are functionally and epigenetically totipotent cells.

This article is not intended to survey the literature comprehensively. Its focal effort is to define different types of totipotency with the genetic, epigenetic, and biochemical underpinnings in mind, and an initial attempt is made to assign the correct totipotency to the zygote and the early blastomeres. Because of limited research on other mammals, this article mainly concerns mouse data, and species will be clearly specified whenever data from other species are used.

## Three Different Molecular Underpinnings of Totipotency

Totipotency is not well defined so far, and the use of this term causes some confusion in the field [2,5]. Condic tried to introduce another term plentipotency [2] and it is later used by another group [6]; Denker coined the term omnipotency [7], and Morgani and Brickman proposed to extend totipotency to some high-quality embryonic stem cells (ESCs) and induced pluripotent stem cells (iPSCs) [5]. The literature frequently calls the zygote and early blastomeres totipotent. However, the different molecular bases for zygote and blastomere totipotency have not been discerned.

By strict definition, totipotency is the ability of a single cell to develop independently into a healthy organism in a permissive environment. By a less strict definition, totipotency is the potential of a cell to differentiate into any type of cells of the body as well as any cells supporting the development of a mammal, including those of placenta and the extraembryonic membranes [3,8,9]. These loose definitions, as well as the available alternative proposals, largely overlook the genetic, epigenetic, and biochemical underpinnings of totipotency. With the molecular underpinnings considered, the term totipotency connotes three fundamentally different concepts: (1) genetic totipotency, referring to the genetic integrity (or contents) of a nucleus or a cell irrespective of the functional status of the genetic materials, active or inactive; (2) the epigenetic (or functional) totipotency, which is the genetic competency (or active status) of a cell with its totipotent genetic determinants active; or (3) the reprogramming capacity of a cell toward epigenetic totipotency, which is the biochemical competency of a cell independent of its genetic compositions and epigenetic status.

Although some sperm proteins may have impact on development [10], the totipotent reprogramming activity is generally from the oocyte factors because an enucleated oocyte without fertilization can reprogram an implanted fully differentiated nucleus to totipotency and give birth to a healthy animal (see discussion in The Zygote Has the Capacity for Reprogramming to Totipotency section, as well as Box 1 and Fig. 2). In other words, the oocyte has the full totipotent reprogramming activity without any contribution from the sperm. For this reason, we may call the third category maternal totipotency as well. The maternal reprograming factors may be proteins and/or RNA from the oocyte reserves. Due to its independence of genetic and epigenetic components, totipotent reprogramming activity in the form of reserve proteins and/or RNA is not sustainable and cannot be captured or maintained in cell culture. The former two concepts are the nuclear features, while the third one concerns mainly the cytoplasmic capacity. A genetically totipotent cell may not be so epigenetically. Totipotent reprogramming factors may be different from those for maintenance of the epigenetically totipotent status. For example, Oct4, a critical factor for the maintenance of embryonic pluripotency and possibly for totipotency, is not required for establishment of totipotency, or for induction of embryonic pluripotency [11]. With the different concepts of totipotency defined above, the relevance of each of these concepts to the zygote is discussed below.

## Genetic Totipotency Is Not Specific to the Zygote

Before animals were first cloned from nuclei of the fully differentiated cells of frogs in the 1960s [12–14], it was posited that cells continuously lose some genetic determinants over the course of development and become permanently restricted in developmental potentials [15]. Only the germ line cells were thought to retain a complete set of the genetic constituents [12,16]. In contrast, a then competing theory, the principle of nuclear equivalence, espoused the notion that a fully differentiated cell contains exactly the same genetic materials as does a blastomere or the zygote, and therefore retains the complete genetic constituents required for development to a healthy individual [17]. In line with this latter principle, many animals of different species have eventually been cloned each from a fully differentiated nucleus after its transfer into an enucleated oocyte (see Box 1 and Fig. 2), providing a clear evidence that a fully differentiated nucleus is still genetically totipotent [12–14,18–21]. This concept has later been corroborated by induction of various somatic cells to pluripotent stem cells (PSCs) via ectopic expression of reprogramming factors [22–24], not only in the form of sustained integrating viral vectors [25,26] but also in the forms of the ephemeral synthetic mRNA [27], recombinant proteins [28], or transient vectors [29–31].

Box 1. **Somatic Cell Nuclear Transfer and Its Applications**

Somatic cell nuclear transfer (SCNT) refers to a technology that a nucleus of a somatic cell is transferred into an oocyte or egg, whose nucleus is removed (enucleated oocyte) or inactivated before implantation of the somatic nucleus (Fig. 2).

The reconstructed egg or embryo by SCNT can then be cultured in vitro to study early development, reprogramming, genetics, biochemistry, and epigenetics.

SCNT plays essential roles in demonstrating both genetic totipotency and the totipotent reprogramming activity. SCNT was originally developed by King and Briggs to test whether a differentiated nucleus still retains full developmental potency [15,116]. Using SCNT, John Gurdon unambiguously showed that a fully differentiated nucleus can give rise to a mature animal and is therefore genetically totipotent [12,13]. At the same time, this shows that the cytoplasm of an oocyte has totipotent reprogramming capacity.

In mammals, the reconstructed embryo can also be transferred into a pseudopregnant foster mother to study development, as well as to clone many mammals. SCNT is responsible for the cloning of the first large animals first from a nucleus of the blastomere [117], and eventually from a fully differentiated nucleus, giving birth to the famous sheep, Dolly [18]. Many different mammals have been cloned by SCNT such as mice, cow, dog, and pigs. The latest cloned species by SCNT is the macaque monkey in 2018 [118].

SCNT is also used to establish pluripotent embryonic stem cells (ESC) lines, that is, therapeutic cloning. In this case, the reconstructed embryo is allowed to grow in vitro to the blastocyst stage. The inner cell mass of the cloned blastocyst is then used as a source to generate nuclear transfer ESC (NT-ESC) lines of somatic origins. NT-ESC lines have been established with fibroblasts as nucleus donors from rhesus monkey [119] and human [120].

Clearly, genetic totipotency does not apply to cells that have lost their genomes such as enucleated oocytes, enucleated zygotes, enucleated blastomeres, mature red blood cells, and platelets. Many individual genes are essential for genetic totipotency since we have seen embryonic lethality when a specific gene is knocked out. Apparently, not every gene is required for genetic totipotency because we have generated so many mice with a specific gene knocked out.

Of note, polyploid cells may be compromised in genetic totipotency, as supported by the fact that cells of a tetraploid embryo rarely contribute to the development of the embryo proper [32,33].

A mammalian haploid genome seems deficient in genetic totipotency. First, no haploid mammals have ever been observed although haploid invertebrates are well known [34]. Features of haploid ESCs also provide some insights into the totipotency of the mammalian haploid cells. Mouse and monkey haploid ESCs have been established, but the karyotypes of those cells are very unstable [35,36]. They undergo diploidization in the culture and during differentiation both in vitro and in vivo. The human haploid ESCs are genetically more stable in culture and during differentiation, but other factors may impair the totipotent potential of human haploid cells. These may include deficiency in parental imprinting, DNA and RNA levels (dosage imbalance), cell size, mitochondrial abundance, and skewed expression ratio of X-linked and autosomal genes [34].

Most aneuploidy cells may lose genetic totipotency. Aneuploidy mainly originates from nondisjunction of chromosomes/chromatids during meiosis I and II [37]. Their developmental potentials can be inferred from human clinical data. First of all, trisomies of all chromosomes with the exception of chromosome 1 have been reported in spontaneous abortions that occur between 6 and 20 weeks of gestation, but trisomy is restricted to a few chromosomes in later stages, that is, stillbirths and live births [38], indicating that trisomy in most chromosomes causes early developmental arrest. Second, the incidence of aneuploidy drops significantly over developmental stages with rates of 25%, 5%, 0.34%, and 0.3% for oocytes, first trimester (5–12 weeks), 13–40 weeks, and after 40 weeks, respectively [38,39]. This, again, indicates that the majority of aneuploid embryos arrest at very early stages.

The genetic totipotency of monosomy appears to be impaired more severely because monosomies all abort before being clinically recognized. Theoretically, if monosomies and trisomies would have the same developmental potentials they should have the same incidence because they are the results of reciprocal events at meiosis [38]. Interestingly, some aneuploidy may have very little impact on totipotency. For example, 0.1% to 0.2% newborn male infants have the genotype of 47, XXY [40]. Many XXY individuals do not even notice their genetic differences in their entire lives.

It is now widely accepted that most cells within an individual have the same genetic makeup as that of the zygote and other early embryonic cells within the cleavage-stage embryos. Therefore, all the different types of diploid cells of our bodies, undifferentiated or differentiated, embryonic or somatic, are genetically totipotent. Being a general feature, genetic totipotency, however, is not a characteristic that uniquely distinguishes a zygote from any normal diploid somatic cell.

## The Zygote Is Not in an Epigenetically Totipotent State

Like embryonic pluripotency of a cell, totipotency of a cell should be defined by the cellular function [5]. Pluripotency has been defined as the potential of a cell to differentiate into any type of cells in a developing embryo proper, and eventually into any type of cells in an adult mammal [41,42]. The conventional mouse pluripotent ESCs do not differentiate into the cells of a placenta. It is commonly held that a pluripotent embryonic cell has limited potential to differentiate into extraembryonic cells [41–45] although an enigmatic observation is that the conventional human ESCs and their mouse counterparts, epiblast stem cells representing a later stage of development than the mouse ESCs, differentiate in vitro into extraembryonic tissues when treated with BMP4 [46,47]. Similarly, functional totipotency is the potential of a cell to differentiate into any type of both the embryonic and the extraembryonic cells during embryogenesis. However, unlike some lower animal zygotes, a mammalian zygote does not differentiate directly into any lineage (see detailed discussion in A Revised Model section about the differentiation ability of the mammalian zygotes). The first lineage differentiation is several cell divisions away from the zygote.

Each type of cells has its own specific transcriptome [48,49]. The function of a cell is generally governed by the cell-type-specific transcriptional program. Underlying any cell-type-specific transcriptional program is its defined epigenetic landscape [50,51]. Therefore, functional totipotency can be called transcriptional or epigenetic totipotency as well.

The zygotic totipotency in the literature should refer to the second concept of totipotency, epigenetic or functional totipotency of a cell described above. By definition, a zygote should have the defined totipotent epigenetic landscape and the corresponding unique totipotent transcriptional program.

The greatest issue with calling the mammalian zygote totipotent is that there is little transcription of its own in zygote. The transcriptome of the zygote is literally that of the oocyte, or a subset of the oocyte's for the later stage of the zygote [52]. The transcriptome of the blastomeres from the early two-cell-stage embryos is still predominantly that of oocyte, and a significant amount of transcripts at the middle two-cell embryo are of maternal in origin [52]. The zygote genome has to be activated to become ready for development or differentiation. Zygotic or embryonic genome activation (ZGA or EGA) is a multiple step process [53,54]. In mice, it initiates at the end of the zygote [55], but the major EGA is at the two-cell stage [54]. At the same time, the maternal mRNA and proteins have to be cleared up for development to start [56]. Clearance of maternal messages in mice is still an ongoing process in the two-cell stage [53]. Therefore, a totipotent state may not be realized transcriptionally before the completion of EGA.

Underlying the general absence of transcription is the incompetence of zygote chromatin for transcription. A permissive chromatin for the general transcription and the transcription of housekeeping genes is not fully available yet before EGA starts, let alone a permissive chromatin for transcription of the totipotent genes.

Totipotent markers are not established yet, but it is suggested that early coexpression of markers for both of the first two lineages [the pluripotent lineages and trophectoderm (TE)] may mark the still uncommitted totipotent cells [5,9]. This is analogy to the bivalent epigenetic markers in PSCs, in which the existence of both activating and repressive marks in a promoter poises a developmental gene for quick expression upon initiation of differentiation [57]. Therefore, the earliest indiscriminate expression of pluripotent markers could potentially indicate an undifferentiated totipotent state, at least for a late stage of totipotency in an embryo. Among these, Oct4 is a good candidate totipotent or “primed” totipotent marker since it is ubiquitously expressed in all blastomeres of early embryo [58,59]. Embryonic expression of *Oct4* is repressed before the eight-cell stage [11,60,61].

Although Nanog is considered the authentic marker for epiblasts (EPI), it is first expressed before separation of outer cells, and is not restricted to the inner cells when inner cells start to emerge [58]. The embryonic (or zygotic) expression of *Nanog* starts only at around morula in mice [62,63]. Cdx2 is an established marker for TE. However, like *Nanog*, *Cdx2* expression is ahead of emergence of TE. Again, like *Nanog*, early *Cdx2* expression is not restricted to the outer cells [58]. Therefore, although unlikely a totipotent marker by itself, initial nondifferential expression of *Cdx2* in all blastomeres with a coexpression of *Oct4* may mark the very late stage of totipotency, a primed totipotency similar to the primed pluripotency. *Cdx2* activation is around 10 h after compaction, slightly later than *Nanog* [58]. Similar to Oct4, both Nanog and Cdx2 are expressed in all blastomeres during early compaction stage before their localization into inner cell mass and TE, respectively [58]. This bivalent expression of lineage markers is in agreement with the developmental plasticity of early outer and inner cells (see discussion in A Revised Model section).

**FIG. 1.**
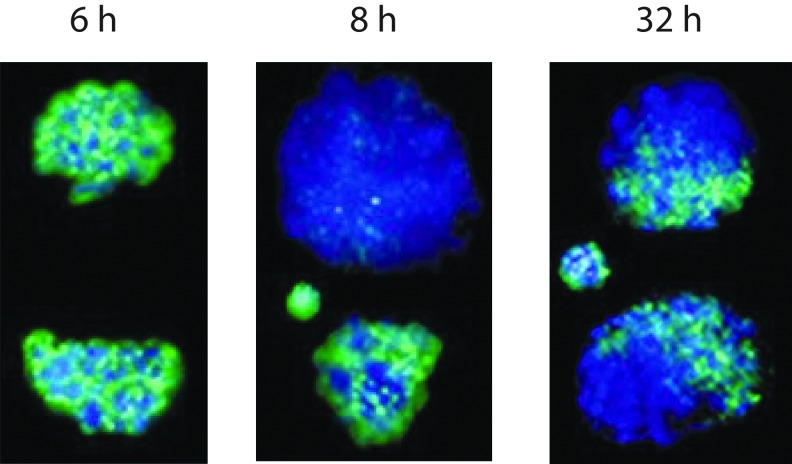
The paternal and maternal genomes are physically separated, under differential reprogramming, and epigenetically distinct in the mouse zygote. *Green*, 5-methylcytosine detected with antibody; *blue*, DNA staining; hours postfertilization are indicated above each image; *Left* and *middle panels*, one zygote in each panel with its two separate pronuclei; *right panel*, a two-cell stage embryo. Small nuclei in the *middle* and *right panels* are polar bodies. Note the extensive demethylation of the paternal pronucleus in the zygote 8 h postfertilization in the *middle panel*, and compartmentalized paternal and maternal chromosomes with differential methylation on cytosines in each of the two-cell blastomeres. Images are courtesy of Thomas Haaf with permission from Nature.

A major function of zygote is epigenetic reprogramming [50,64,65]. To establish totipotency, the epigenetic marks for both maternal and paternal genomes have to be erased first, and then rewritten [66]. A prominent fact about the zygote epigenetics is that the paternal and maternal genomes are dramatically different in epigenetic marks and chromatin structure [4]. For example, they are differentially methylated both in DNA and histones [67]. Furthermore, the two parental genomes are in fact physically separated during the entire zygote life (Fig. 1) [68,69]. Recently, it is found that the paternal and maternal genomes have their own spindles during the first cell division [70]. These data show that the unification of the parental genomes has not been completed yet by the end of zygote. Physical separation of maternal and paternal genomes is still apparent in the two-cell stage although to a lesser degree (compartmentalization) [68] (Fig. 1). Physical separation of parental genomes in the zygote indicates that fertilization is not finished yet at the end of zygote because only complete pronuclear fusion marks the end of fertilization [71]. This physical separation of parental genomes allows differential reprogramming of the two genomes. For example, the paternal genome is already extensively demethylated 8 h after fertilization, but extensive erasure of DNA methylation in the maternal genome is apparent only at the four-cell stage [68] (Fig. 1). In summary, zygote still has two separate parental genomes with different epigenetic landscapes, which are under dramatic and dynamic reprogramming (Fig. 1). A zygote is not in a totipotent state transcriptionally, epigenetically, and functionally.

## The Zygote Has the Capacity for Reprogramming to Totipotency

Oocytes are the most powerful reprogramming vehicle in nature [72,73]. An enucleated oocyte (or with its nuclear destroyed) can reprogram an implanted fully differentiated somatic nucleus to functional totipotency and gives rise to a cloned animal [14,18,19]. Reprogramming activity exists beyond oocytes. *Oct4-GFP* reporter experiments using somatic cell nuclear transfer (SCNT) technology (see Box 1) indicate that reprogramming occurs at cleavage stages [74]. An enucleated zygote can still reprogram an implanted differentiated nucleus to totipotency when the right procedure is used [75]. Upon fertilization of an oocyte, the paternal and maternal chromatin starts epigenetic and transcriptional reprogramming to totipotency by the oocyte factors. This totipotent reprogramming process continues beyond the zygote; even a blastomere from a two-cell stage embryo retains significant capacity for totipotent reprogramming [76,77]. Persistence of oocyte reprogramming activity into the two-cell stage is additionally supported by the generation of mice after injection of a round spermatid into a haploid parthenogenote [78], which is an equivalent to a blastomere of a two-cell embryo.

**FIG. 2.**
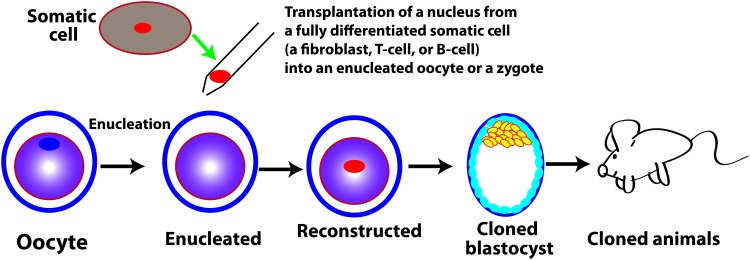
The totipotent reprogramming activity of an oocyte or a zygote is independent of epigenetic status. Note that, like the united sperm and oocyte genomes, an individual nucleus at distinct differentiated epigenetic states (fibroblasts, T cells, or B cells) can be reprogrammed by an enucleated oocyte, which is lacking any of its own nuclear genetic material, to totipotency and gives rise to an animal.

Of note, the totipotent reprogramming activity in oocyte and zygote is independent of their epigenetic status because an enucleated oocyte can reprogram into totipotency the fully differentiated implanted genomes of various origins, including those from fibroblasts [79], cumulus [19], Sertoli cells [80], T cells, B cells [81], and others (Fig. 2). Therefore, the cellular function of a zygote is totipotent reprogramming endowed by the maternal reprogramming factors inherited from its parental oocyte although the reprogramming activity may be attenuated, but the zygote genome is not in a totipotent state transcriptionally, epigenetically, and functionally. Like in the zygote, a blastomere from a two-cell stage embryo may still be in the reprogramming process toward totipotency since it can reprogram an implanted nucleus to totipotency [76,77]. Similar to zygote, the totipotency of a blastomere from the two-cell stage embryo may be largely maternal since it still retains a significant amount of oocyte factors.

## A Revised Model for Cellular Totipotency

**FIG. 3.**
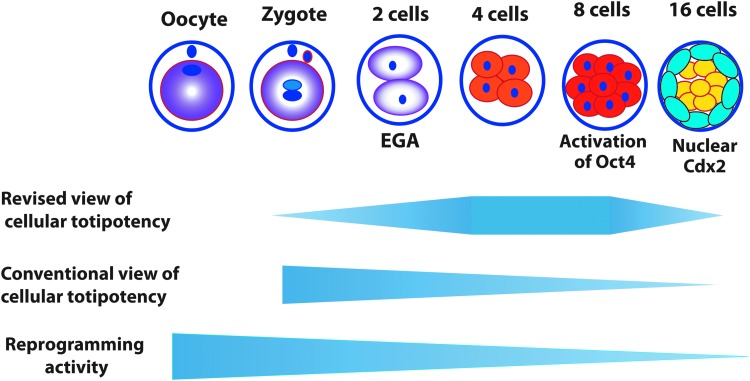
A revised model for reprogramming capacity and differentiation potential of an early embryonic cell in comparison with the conventional view [1]. The schema is based on mouse, in which the epigenetic totipotency may exist from the four-cell to the eight-cell embryo before compaction. The time line for other species may vary. Cytoplasmic *purple* represents the totipotent reprogramming activity of maternal origin. EGA, embryonic genome activation.

In light of discussions above, a modified model describing capacity for cellular totipotency is proposed (Fig. 3). The first totipotent cell should be after the major EGA because only after EGA cell autonomous function can be provided by its own transcription independent of oocyte-derived biochemical factors. The functional aspect of the totipotent cells is manifested by the first differentiation event in embryogenesis. This first differentiation in mammalian life cycle apparently does not occur in zygote, or at the two-cell stage of embryogenesis. The first cellular differentiation during mouse embryogenesis is likely at the morula stage, at which point the first two types of cells, embryoblasts (inner blastomeres) and TE (outer blastomeres) begin to emerge. Therefore, totipotent cells should be those immediately before this first differentiation. To sum up, a totipotent state should be somewhere between the completion of the major EGA and the separation of the first two lineages in embryogenesis.

In mice, the separation of the first two lineages is initiated by polarization of individual blastomeres when the embryo compacts at the late eight-cell stage, and the subsequent formation of inner and outer layer of cells after the 16-cell stage [58]. It is widely regarded that blastomeres within an embryo are generally uniform in morphology, size, cellular polarity, cell positioning (outside and inside), and developmental potential before compaction [82]. Thus, the totipotent cells in mice may exist at the four-cell and early eight-cell stages, at the latter point of which the embryo compaction still does not occur.

In addition to the discussion above, recent single-cell RNA-seq of mouse embryos provides support for the earlier limit for autonomous totipotency. Mouse blastomeres at the two-cell embryos still feature oocyte transcripts, and normal levels of biallelic expression, that is, embryonic expression, are reached only at the four-cell stage [52]. This result indicates that blastomeres at the two-cell stage rely on maternal RNA to function, while a blastomere at the four-cell stage starts to function on its own transcripts.

The placement of the later limit of epigenetic totipotency is indirectly supported by the fact that no blastomere of the 16-cell mouse embryos is irreversibly committed to either fate of the first two lineages: inner cell mass and TE. Although the separation of the inner and outer cells becomes visible at the 16-cell stage morphologically (distinct morphologies for inner and outer cells) and molecularly (differentially marked by Cdx2 and Oct4), either purified inner or outer cells from the 16-cell mouse embryos are able to develop into normal fertile animals when reaggregated as 16 pure outer cells or 16 pure inner cells [83]. The totipotent plasticity of the blastomeres in the 16-cell embryo is further supported by another experiment, in which four identical mice (quadruplets) were generated each from a single outer blastomere from the same embryo using tetraploid complementation [84]. Outer blastomeres in the 16-cell embryo are destined to form extraembryonic tissues, but this experiment indicates that it can take the other developmental path to form an animal, which is the function of the inner cells. This plasticity indicates that mouse blastomeres at the 16-cell stage still retain some degree of totipotency, but this plastic developmental potential is lost at the 32-cell stage [83]. In human, even the TE cells in the full blastocysts are not irreversibly committed to TE, and can become EPI fate [6,85].

The time of paternal *Oct4* activation during early embryogenesis provides a support for the proposed placement of totipotent state above. The marker for totipotency is lacking partly because embryonic totipotency and maternal totipotency have not been distinguished before this essay. Oct4 may be a shared marker for totipotency and embryonic pluripotency. Unlike other embryonic pluripotency marker, embryonic *Oct4* activates earlier and are expressed uniformly in all blastomeres up to 32-cell embryo [59], while early Nanog is more mosaic. Mouse *Oct4* is activated at the eight-cell stage (Fig. 3) as demonstrated by mRNA in situ hybridization [60], immunohistochemistry [61], and *Oct4:GFP* reporter detection [11]. Allele-specific analysis indicates that paternal *Oct4* is silenced before four-cell stage, and is activated at around the four-cell to eight-cell stages [86]. Interestingly, Plachta et al. show that Oct4 may be heterogeneous in function in eight-cell embryo although it is expressed in every blastomere [87]. In some cells, Oct4 binds to chromatin more stably (more functional), but in other cells Oct4 dissociates from chromatin easily (less functional). Blastomeres with more functional Oct4 tend to undergo division asymmetrically and give rise to one outer and one inner cell. This does not mean these blastomeres are committed cells. This is because even the products of this asymmetrical division, the inner cells and outer cells at the 16-cell stage embryos, are still plastic in totipotency (see discussion above).

The activation time of differentiation markers for the first embryonic lineage, TE, may provide a reference, although not direct markers, for the developmental placement of totipotent cells proposed here. Elevated nuclear expression of the transcription factor Cdx2 in the still plastic outer cells of the morula embryo represents the earliest events in lineage specification [88–90]. Unequivocal nuclear Cdx2 in mice is detected in the prospective TE cells only after the fourth embryonic cell division (the cell division from cells of the eight-cell embryo to generate the 16-cell embryo) [59,88], indicating that totipotent cells in mice may exist before the fourth cell division.

Cleavage-stage blastomeres are generally regarded as being totipotent [3,91–93]. Totipotency of blastomeres rather than zygote has been demonstrated experimentally. A single blastomere from a two-cell embryo gives rise to fertile adult mice [92]. Single blastomeres isolated from a four-cell or eight-cell sheep embryo can develop into lambs [94]. Both individual blastomeres after separated from the same two-cell stage embryo can develop into live animals (monozygotic twins) for mice [95,96], sheep [97], and rat [98]. Four identical calves were generated each from an individual blastomere isolated from the same four-cell bovine embryo [99], and three sheep (triplets) were generated each from one individual blastomere isolated from one single four-cell embryo [94]. In humans, each of the four individual blastomeres from the same embryo can develop into an expanded blastocyst, indicating an individual capacity of each ¼ blastomere to contribute to both of the first two embryonic lineages, the inner cell mass and TE [91].

The model further emphasizes that a mammalian life begins with reprogramming of the united sperm and oocyte genomes, not with differentiation. Since it has been frequently stated that a zygote differentiates into all types of cells [3,4,11], it is critical to set the record straight. Mammalian differentiation is a property of stem cells or progenitor cells. With “totipotent” being an inappropriate defining word for zygote, the associated term “stem cell” does not belong to zygote either. First, zygote has no ability for self-renewal, one of the two basic features of stem cells [100]. A zygote does not divide to become two zygotes because of ongoing dramatic nuclear reprogramming during the early cleavage stage. A zygote divides to become inevitably two blastomeres of the two-cell embryo. Second, a zygote does not differentiate, the second essential feature of stem cells [100]. The entire zygote genome is literally inactive. An inactive genome cannot differentiate and needs to be reprogrammed to totipotency before it can start to differentiate. This reprogramming process may last for several cell cycles depending on species. In the case of mice, the totipotent reprogramming may be complete before the eight-cell stage.

How do we have the concept of zygote differentiation for mammalian embryogenesis? The characterization of “differentiation” for the mammalian zygotes is a preconceived notion based on the studies of early embryogenesis of some lower animals. In lower animals such as *Caenorhabditis elegans* and *Drosophila*, oocytes and zygotes are polarized [101,102]. For example, due to polarized localization of fate determinants in oocyte and zygote, the first cleavage in *C. elegans* gives rise to the AB and P1 blastomeres, which specify anterior and posterior axis, respectively. However, no polarized localization of specification determinants, including mRNA and proteins in mammalian oocytes and zygotes, plays any role in mammalian development [103,104]. Experimental data further show that each blastomere of the two-cell embryo contributes to both of the first two embryonic lineages [105], indicating a lack of embryo polarity at the two-cell stage as well. Even the individual blastomeres of the mouse four-cell embryo contribute impartially to both of the first two lineages [106]. An apicobasal cellular polarity is only seen late at the eight-cell stage, and visible embryo polarity in mammals is established only at the blastocyst stage [107]. Therefore, we cannot simply apply the concept of zygote “differentiation” in some lower animals to mammalian embryogenesis. The development in these lower animals heavily relies on the maternal determinants, and some lower animals may not have totipotent stem cells because “differentiation” occurs in zygote already by the polarized localization of cell fate determinants inside the oocyte and zygote.

## Conclusions and Prospects

This essay systematically defines, for the first time, three distinct types of totipotency: genetic, epigenetic, and nonsustainable biochemical ones. Every normal diploid cell is of genetic totipotency; epigenetic totipotency may exist in embryonic cells immediately before the separation of the first two embryonic lineages. They may be blastomeres in the four-cell and eight-cell embryos of mice. Zygote uniquely retains most of the totipotent reprogramming activity of the oocyte. Zygote is in the transition from maternal to embryonic totipotency.

Totipotency should be a term to define a cell. However, an embryo at any stage represents a special moment of an individual life. The elusive use of totipotency for the zygote may be because the zygote is special in that it is both a single cell and regarded as an embryo by most scientists. As a cell, zygote is (1) genetically totipotent, but this term does not distinguish it from other undifferentiated and differentiated cells, and (2) capable of reprogramming its own as well as an implanted genome to epigenetic totipotency, but (3) the zygote is not in the state of totipotency epigenetically, transcriptionally, and functionally. As a one-celled “embryo” although it is suggested that it is not an embryo yet [71], the zygote is a critical starting point of an animal life, but its ability to develop into an animal is endowed by its totipotent reprogramming activity from the maternal factors (maternal proteins and RNA). Establishment of distinct concepts of maternal and autonomous epigenetic totipotency will benefit further investigation into these two distinct totipotent activities.

The revised totipotency model proposed here has practical significance. This model predicts that we may be able to capture the totipotent cells in cell culture as we have achieved with the first three embryonic lineages: pluripotent ESCs representing the EPI from both mice [42,43] and humans [41], trophoblast stem cells [108] representing the TE from mice [109] and humans [110], and the extraembryonic endoderm stem cells for the primitive endoderm of mouse [111,112]. Recently, extended pluripotent stem (EPS) cells have been captured in culture for both humans and mice [113]. EPS cells contribute to both embryonic and extraembryonic tissues. However, the transcriptome of EPS cells is different from that of any PSCs and embryonic cells although EPS cells share some transcriptional signatures of the eight-cell embryos.

However, it is impossible to perpetuate or proliferate a zygote in cell culture because the zygote does not have a stable active epigenetic status, and almost completely relies on the ephemeral maternal factors (eg, proteins, mRNA, and microRNA) to function. Similarly, we may not be able to capture the blastomeres of a two-cell embryo in cell culture because such blastomeres still rely on the maternal factors to function and their epigenetic and transcriptional states are still unstable and very dynamic. It is reported that cells expressing murine endogenous retrovirus activity, a characteristic of the two-cell embryos, transiently exist in mouse PSC culture in a very small portion (2C-like cells) [114]. These “2C-like” cells in PSC culture cannot be expanded independently and the purified 2C-like cells return to the normal PSC state. The “2C-like” cells seem to be in a state of repression of global protein synthesis independent of mitosis [115]. The expandable EPS cells have no molecular signature of the “2C-like” cells [113].
